# Inhibition of hepatocyte nuclear factor 1β contributes to cisplatin nephrotoxicity via regulation of nf‐κb pathway

**DOI:** 10.1111/jcmm.16316

**Published:** 2021-01-29

**Authors:** Yan Zhang, Jielu Hao, Zijun Du, Peiyao Li, Jinghua Hu, Mengna Ruan, Shulian Li, Yuanfang Ma, Qiang Lou

**Affiliations:** ^1^ Joint National Laboratory for Antibody Drug Engineering Henan University Kaifeng China; ^2^ Department of Biochemistry and Molecular Biology Mayo Clinic Rochester MN USA; ^3^ Department of Nephrology Changzheng Hospital Naval Medical University Shanghai China

**Keywords:** cisplatin nephrotoxicity, Hepatocyte nuclear factor 1β, NF‐κB

## Abstract

Cisplatin nephrotoxicity has been considered as serious side effect caused by cisplatin‐based chemotherapy. Recent evidence indicates that renal tubular cell apoptosis and inflammation contribute to the progression of cisplatin‐induced acute kidney injury (AKI). Hepatocyte nuclear factor 1β (HNF1β) has been reported to regulate the development of kidney cystogenesis, diabetic nephrotoxicity, etc However, the regulatory mechanism of HNF1β in cisplatin nephrotoxicity is largely unknown. In the present study, we examined the effects of HNF1β deficiency on the development of cisplatin‐induced AKI in vitro and in vivo. HNF1β down‐regulation exacerbated cisplatin‐induced RPTC apoptosis by indirectly inducing NF‐κB p65 phosphorylation and nuclear translocation. HNF1β knockdown C57BL/6 mice were constructed by injecting intravenously with HNF1β‐interfering shRNA and PEI. The HNF1β scramble and knockdown mice were treated with 30 mg/kg cisplatin for 3 days to induce acute kidney injury. Cisplatin treatment caused increased caspase 3 cleavage and p65 phosphorylation, elevated serum urea nitrogen and creatinine, and obvious histological damage of kidney such as fractured tubules in control mice, which were enhanced in HNF1β knockdown mice. These results suggest that HNF1β may ameliorate cisplatin nephrotoxicity in vitro and in vivo, probably through regulating NF‐κB signalling pathway.

## 1 INTRODUCTION

Cisplatin is one of the most commonly used chemotherapy drugs; however, the severe side effects such as nephrotoxicity limit its dose administered in cancer patients. During cisplatin‐induced acute kidney injury (AKI), renal tubular cells are extremely vulnerable to damage because of high drug accumulation. Cisplatin nephrotoxicity is characterized by cell apoptosis and inflammation.

The nuclear factor kappa‐light‐chain‐enhancer of activated B cell (NF‐κB) signalling pathway is important for regulating cell apoptosis. The NF‐κB p50/p65 heterodimer is mainly located in cytoplasm. Upon cisplatin stimulation and IκB kinase activation, the NF‐κB translocates to nucleus and transcriptionally activates target gene expression such as interleukin‐1β (IL‐1β) and tumour necrosis factor α (TNF‐α).^1^ Cisplatin has the ability to induce NF‐κB phosphorylation in HEK293 cells and further decrease the secretion of pro‐inflammatory cytokines such as IL‐1β and TNF‐α.^2^ Cisplatin also can increase NF‐κB expression in kidney tissues of rat.^3^ During cisplatin‐induced mouse renal injury, NF‐κB activation, IκBα phosphorylation and p65 protein nuclear translocation were observed. Moreover, the P53 protein level, caspase 3/9 and the poly (ADP‐ribose) polymerase (PARP) cleavage were also increased.^4^, ^5^ The transcriptional inhibition of NF‐κB was reported to be able to ameliorate cisplatin‐induced AKI.^6^ However, the upstream genes regulating NF‐κB transcriptional activation during cisplatin nephrotoxicity are still not fully understood.

The transcription factor HNF1β (hepatocyte nuclear factor 1 homeobox B) was found to be abundantly expressed in kidney and pancreas,^7^ but not in brain and heart tissues. HNF1β was expressed higher in kidney than in lung and liver tissues.^8^ Early reports have linked HNF1β regulation to kidney cystogenesis and development.^9^ Patients with HNF1β mutations often complicated with diabetes, renal cysts and loss of kidney function.^10^, ^11^, ^12^, ^13^ Upon exposure to TNF‐α and interferon‐γ (IFN‐γ), the proximal tubular cells exhibited inhibited transcriptional activity of HNF1β and dysfunction of mitochondria.^14^ Thus, the transcriptional activity of HNF1β may be related to the inflammatory response of renal tubular cells.

HNF1β was involved in regulating various pathological processes. HNF1β knockdown in proximal tubular HK2 (human kidney 2) cells promoted the epithelial‐to‐mesenchymal transition.^15^ HNF1β positively regulated the proliferation and tubulogenesis of normal kidney proximal epithelial cells (NRK‐52E).^16^ In our previous study, HNF1β was found to play protective role in cisplatin‐induced tubular cell apoptosis in vitro.^17^ However, in Wilms' tumour‐derived G401 cells, HNF1β was reported to decrease cell proliferation and migratory abilities and increase apoptotic rate no matter through overexpression or small interfering RNA‐silencing experiments.^18^ Therefore, the role and mechanism of HNF1β in cisplatin‐induced apoptosis still need further analysis.

In this study, the in vivo role of HNF1β in cisplatin nephrotoxicity was examined. Moreover, the regulatory function of HNF1β on NF‐κB activation was analysed.

## 2 MATERIALS AND METHODS

### 2.1 Regents, cell lines and animals

Rat kidney proximal tubular cells (RPTCs) were originally obtained from Dr Hopfer (Case Western Reserve University) and were cultured in DMEM/F12 medium (12400024; Gibco) containing transferrin (5 μg/mL) (T8158; Sigma), insulin (5 μg/mL) (I9278; Sigma), epidermal growth factor (EGF; 1 ng/mL) (E9644; Sigma), dexamethasone (4 μg/mL) (D1756; Sigma), 10% foetal bovine serum (FBS) (PAN‐Seratech) and 1% antibiotic‐antimycotic (15240062; Thermo Fisher Scientific). RPTC HNF1β knockdown and scramble cell lines were constructed in our previous study.^17^ Lipofectamine 3000 Transfection Regent (L3000008) was purchased from Thermo Fisher Scientific Company.

About 8‐week‐old C57BL/6J male mice were purchased from Beijing Vital River Laboratory Animal Technology Co., Ltd (China). Mice were acclimated for 7 days before formal experiments and were housed in groups of four animals per cage. Animal experiments were performed in accordance with the approval of the Ethics Committee for Experimental Animals in Henan University (Approval Number: DWLL20200102).

### 2.2 HNF1β interference mouse model construction

The HNF1β‐specific shRNA plasmid was purchased from Qiagen Company (Catalogue Number: 336312 KR45380H, USA). The target sequence of si‐HNF1β was 5’ GTGTAACAGGGCAGAATGTTT 3’. The construction of HNF1β knockdown mice was performed as described in previous studies with mild modifications.^19^, ^20^, ^21^ Briefly, the HNF1β scramble or short hairpin RNA (shRNA) was mixed with PEI (Polyethyleneimine, 765090; Sigma) at a ratio of 1:2. C57BL/6 mice aged about 8 weeks were injected with 40 μg HNF1β scramble or shRNA plasmids by tail vein. Mice were randomly divided into three groups: Scramble group in which mice were treated with a non‐specific shRNA for 24 hours (n = 6); and shRNA‐HNF1β groups in which mice were treated with specific HNF1β shRNA for 24 hours (n = 8) or 72 hours (n = 8).

### 2.3 Cisplatin‐induced AKI mouse model construction

To construct a C57BL/6 mouse model of acute kidney injury induced by cisplatin, C57BL/6 male mice aged 6‐8 weeks were randomly divided into four groups: vehicle, mice treated with phosphate‐buffered saline (PBS) (n = 8); cisplatin 1d, mice treated with cisplatin for 1 day (n = 8); cisplatin 2d, mice treated with cisplatin for 2 days (n = 8); and cisplatin 3d, mice treated with cisplatin for 3 days (n = 8). Mice in each group were intraperitoneally injected with 30 mg/kg cisplatin, and the cervical venous blood and kidneys of the mice were collected at 24, 48 and 72 hours after modelling, respectively.

To further evaluate the impact of HNF1β interference on cisplatin‐induced nephrotoxicity, C57BL/6 male mice aged 6‐8 weeks were randomly divided into four groups as follows: shRNA‐scramble group, shRNA‐HNF1β group, cisplatin with shRNA‐scramble group and cisplatin with shRNA‐HNF1β group. Mice were given tail injection of 40 μg HNF1β‐interfering plasmids. An hour later, these mice were intraperitoneally injected with PBS or 30 mg/kg cisplatin for 72 hours. The mice were anaesthetized by intraperitoneal injection of 2% chloral hydrate (0.015 mL/g; Esite Biotechnology, China). The mouse kidneys were extracted after modelling for further analysis. The mouse serum was separated from venous blood samples by centrifugation at 12 000 *g* at room temperature for 5 minutes. Renal function was assessed by measuring serum creatinine (0420‐500; Stanbio) and blood urea nitrogen (BUN) (0580‐250; Stanbio) following Jaffe's and GLDH reactions.

### 2.4 RPTC culture

The RPTC HNF1β‐negative control cells and knockdown cells were treated with 20 μmol/L cisplatin with or without 50 μmol/L NF‐κB pathway inhibitor TPCA (S2824; Selleck Chemicals) for 24 hours. Then, Hoechst 33342 (10 μg/mL; Beyotime Biotechnology, China) was used for nuclear staining. The apoptotic cells were then visualized under a Nikon Inverted Microscope Eclipse Ti‐E (Nikon, Japan) and counted from five randomly selected fields by ImageJ software.

### 2.5 Immunofluorescence

The RPTC HNF1β‐negative control (NC) cells and knockdown (KD) cells were treated with or without 20 μmol/L cisplatin. Briefly, 1 × 10^5^ cells were seeded into 35‐mm dishes containing slides with a diameter of 1 cm and then were cultured until 80% confluence. After 24 hours of treatment with 20 μmol/L cisplatin, the slides were washed with PBS and penetrated with cold methanol for 10 minutes. The slides were incubated with anti‐HNF1β antibody (1:50, 12533‐1‐AP; Thermo Fisher Scientific) or anti‐phospho‐P65 antibody (1:1600, 3033; Cell Signaling Technology) overnight at 4°C and then incubated with Alexa Fluor 488–labelled secondary antibody (1:1000, Thermo Fisher Scientific, USA) for 1 hour at room temperature. Cell nucleus was stained with DAPI (P36941; Thermo Fisher Scientific) for 10 minutes. The images were acquired by Nikon Inverted Microscope Eclipse Ti‐E (Nikon, Japan), and the nuclear translocation was quantified using ImageJ software as previously described.^22^

### 2.6 TUNEL staining

The cells with a confluence of up to 80% were extracted from the cell incubator, namely RPTC HNF‐1 NC cells and RPTC HNF‐1 KD cells. The cells were digested by trypsin (1004GR025, BioFroxx, Germany) and collected. The cells were evenly spread to the 24‐well plate according to the density of 8 × 104/well and then cultured for 24 hours in the cell incubator (Model: 3111; Thermo Scientific, USA) at 37℃ and 5% CO_2_. The next day, the cells were grouped as follows: NC or KD cells treated with or without 20 μmol/L cisplatin, NC or KD cells treated with cisplatin and TPCA‐1 (S2824; Selleck Chemicals), and NC or KD cells treated with TPCA‐1 only. Cells were rinsed with PBS for 2‐3 times and fixed at room temperature by 4% paraformaldehyde for 1 hour. After washed with PBS, cells were blocked with 3% H_2_O_2_ solution (323381; Sigma) at room temperature away from light for 10 minutes. Cells were permeabilized with Triton X‐100 (T8200; Solarbio) at concentration of 0.05% (v/v) for 2 minutes on ice and incubated with TUNEL reaction buffer (P48307791‐131; Roche) at 37℃ for 1 hour away from light. The cell nuclei were stained with Hoechst 33342 (10 μg/mL) for 2 minutes at room temperature. The cells were observed under a Nikon Inverted Microscope Eclipse Ti‐E (Nikon, Japan).

### 2.7 Cytoplasm and nucleus protein extraction

RPTC HNF1β NC or KD cells were treated with or without 20 μmol/L cisplatin for 24 hours, and then, the cells were digested by SDS (S8010, Solarbio) lysis buffer and were centrifuged at 500 *g* for 5 minutes. The centrifuged precipitates were further used for isolation of cytoplasm and nucleus proteins by NE‐PER nuclear and cytoplasmic extraction reagents (78833, Thermo Scientific).

### 2.8 Western blot

RPTCs were treated with or without 20 μmol/L cisplatin for 24 hours. Total cell proteins were extracted using SDS lysis buffer containing 1× Protease Inhibitor Cocktail (RF232581; Thermo) and were quantified using BCA Protein Quantification Kit (CW0014; Cwbiotech Company, China). Kidney cortex tissues were homogenized in SDS lysis buffer containing 1× Protease Inhibitor Cocktail at 4°C and then were centrifuged at 15 322 *g* for 5 minutes at 4°C. The supernatants were used for protein quantification. Protein samples were separated using SDS‐PAGE (polyacrylamide gel electrophoresis) and then were transferred onto PVDF membrane (IPVH00010; Immobilon) hydrated with methanol. The membranes were incubated with primary antibodies over night at 4°C and secondary antibodies for 2 hours at room temperature. The primary antibodies were rabbit anti‐HNF1β antibody (12533‐1‐AP; Thermo Fisher Scientific), rabbit anti‐caspase 3 antibody (9662; CST) and rabbit anti‐cleaved PARP antibody (BS7047; BioWord) purchased from Cell Signaling Company. The second antibodies were HRP‐labelled anti‐mouse (AS003; ABdone) or anti‐rabbit IgG antibody (AS014; ABdone). The ECL Chemiluminescence Detection Kit (WBKLS0500; Millipore Company) was used for signal detection. The protein bands were quantified using ImageJ software.

### 2.9 Haematoxylin and eosin (H&E) staining

Kidney cortex tissue sections were placed in a 60℃ oven for 4 hours, dewaxed with xylene in fume hood for 3 times, each time for 10 minutes, and then dehydrated with 100%, 96%, 90%, 80% and 70% concentrations of ethanol for 5 minutes, respectively. After washing with running tap water for 10 minutes, the sections were stained with haematoxylin for 30 seconds, decolorized with acid ethanol (1% HCl and 99% medical alcohol) for 1‐2 seconds and then stained with eosin for 40 seconds. The sections were observed and photographed under a Nikon Inverted Microscope Eclipse Ti‐E (Nikon, Japan).

### 2.10 Immunohistochemical staining

The dewaxing and dehydrating processes were the same as HE staining. The tissues were blocked with 3% hydrogen peroxide for 20 minutes at room temperature in the dark. Then, the sections were sealed with 5% BSA for about 30 minutes at room temperature and were incubated with primary antibodies against cleaved caspase 3 (9662; CST), HNF1β antibody (12533‐1‐AP; Thermo Fisher Scientific) and phosphor‐p65 (3033; Cell Signaling Technology) at dilution of 1:200 at 4°C overnight. The second antibodies were HRP‐labelled anti‐mouse (AS003; ABdone) or anti‐rabbit IgG antibody (AS014; ABdone). The tissues were stained with 50 μL chromogenic agent diaminobenzidine for 30 seconds and then with haematoxylin for 10 seconds. Photographs were taken under microscope with 200 × magnification.

### 2.11 Statistical analysis

The results of this experiment were all repeated for 3 times, and Prism (GraphPad Software 7) was used for data analysis. *t* Test, one‐way ANOVA and two‐way ANOVA were used for data comparison between groups. The data were expressed as means ± SEM, and *P* < 0.05 was considered as statistically significant.

## 3 RESULTS

### 3.1 Inhibition of HNF1β exacerbated cisplatin‐induced RPTC apoptosis

**FIGURE 1:**
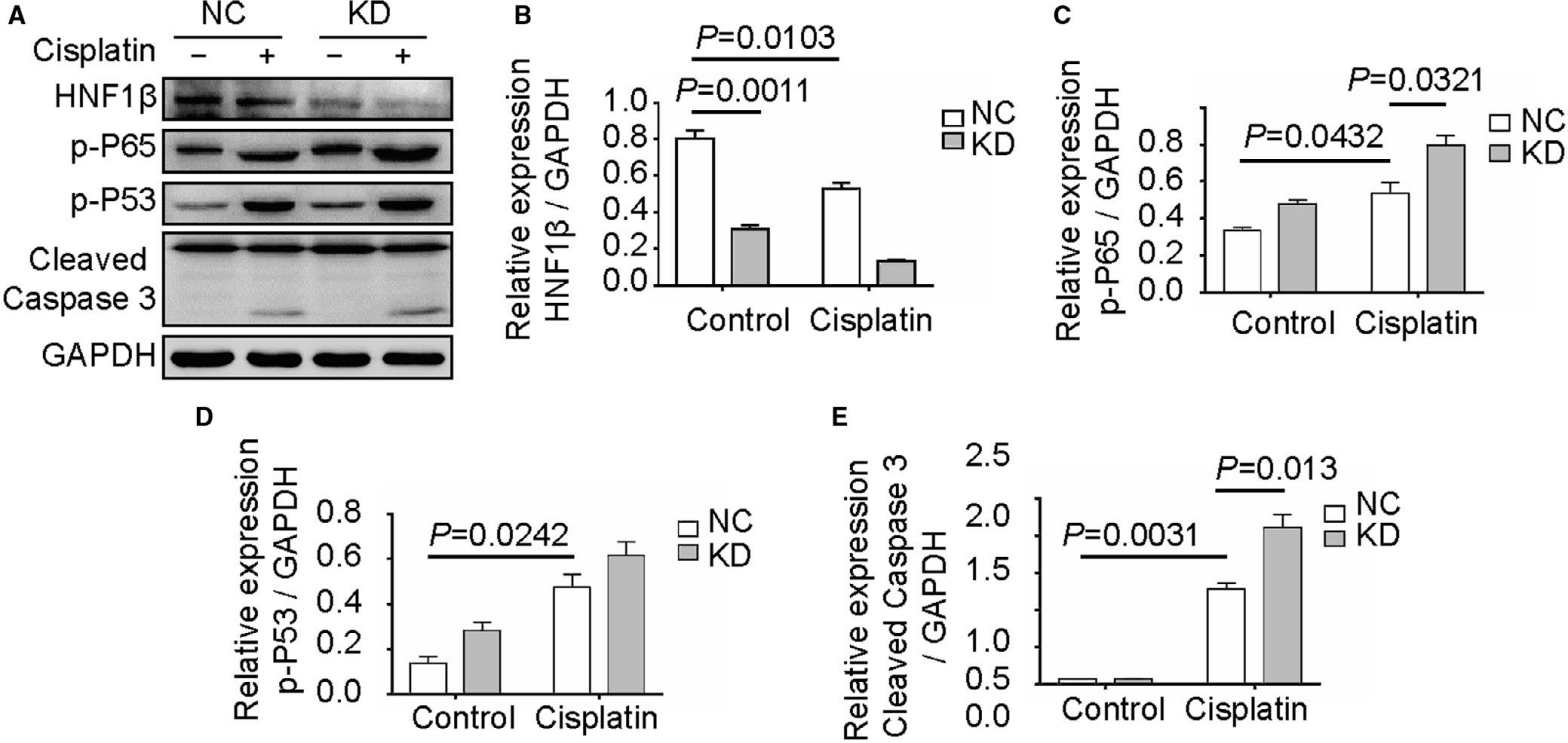
Protein expression affected by HNF1β in RPTCs after treatment with cisplatin. RPTC HNF1β‐negative control (NC) and knockdown (KD) cells were treated with or without 20 μmol/L cisplatin for 24 h. (A) Total proteins were extracted for Western blot analysis of HNF1β, phosphor‐P65, phosphor‐P53 and cleaved caspase 3, in which GAPDH was used as an internal control. (B, C, D, E) Relative quantification and statistical analysis of HNF1β, phosphor‐P65, phosphor‐P53 and cleaved caspase 3 using ImageJ software. *P* < 0.05 was considered as significant difference

We previously determined the protective role of HNF1β in cisplatin‐induced RPTC apoptosis; however, the exact mechanism of HNF1β regulating cisplatin nephrotoxicity is still unclear. In this study, the HNF1β‐NF‐κB signalling pathway during cisplatin‐induced apoptosis was examined. RPTC HNF1β scramble (negative control, NC) and HNF1β shRNA (knockdown, KD) cells were treated with 20 μmol/L cisplatin for 24 hours, and total proteins were extracted for Western blot analysis. HNF1β expression level was down‐regulated to about 65.8% after stimulation of cisplatin (Figure 1A,B). Hoechst staining of RPTC nuclei indicated the cisplatin‐induced apoptosis of HNF1β knockdown cells was significantly increased in comparison with that of RPTC HNF1β NC cells (data not shown).

### 3.2 Inhibition of HNF1β‐induced NF‐κB signalling activity during cisplatin nephrotoxicity

**FIGURE 2:**
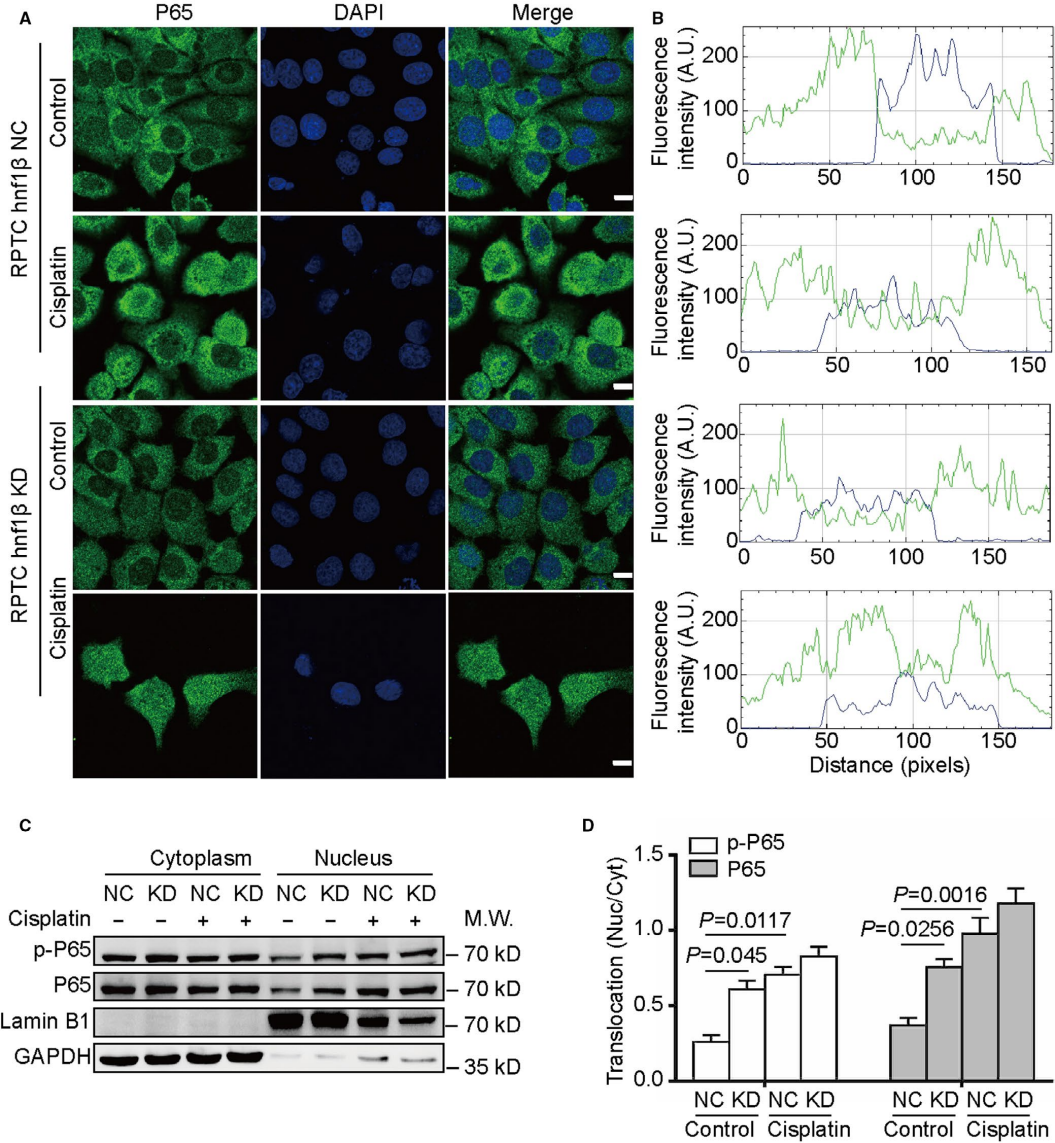
NF‐κB p65 nuclear translocation affected by HNF1β in RPTCs after treatment with cisplatin. RPTC HNF1β‐negative control (NC) and knockdown (KD) cells were treated with or without 20 μmol/L cisplatin for 24 h. (A) Immunofluorescence laser confocal analysis of the green (p65) and blue (nucleus) signals. Scale bars are 10 μm. (B) Fluorescence intensities of the green and blue signals were quantified in arbitrary units (AU) by ImageJ software. (C) Western blot analysis of phosphor‐P65 and total P65 in the cytoplasmic and nuclear fractions of RPTC NC and KD cells. Lamin B1 and GAPDH were used as internal controls of nuclear and cytoplasmic proteins, respectively. (D) The ratios of phosphor‐P65 or total P65 in nucleus and cytoplasm were quantified using ImageJ software

After cisplatin treatment, the phosphorylation of p65 NF‐ĸB and p53 and the cleavage of caspase 3 were induced by 1.62 and 3.43 times, respectively. Compared with NC cells, cisplatin treatment of RPTCs increased p65 NF‐ĸB phosphorylation and caspase 3 cleavage in HNF1β knockdown cells by 1.44 and 1.64 times, respectively (Figure 1A‐E). Moreover, the cisplatin‐induced NF‐κB p65 nuclear translocation in RPTC HNF1β NC and RPTC HNF1β KD cells was examined by confocal analysis (Figure 2A). RPTCs were fixed with ethanol and stained with anti‐HNF1β antibody and FITC‐labelled secondary antibody. The fluorescence intensity was quantified using ImageJ software (Figure 2B). The green signals were mainly found in cytosol in control RPTCs. The ratio of green signal–positive cells was markedly increased after cisplatin treatment. Cisplatin promoted the nuclear translocation of P65 in RPTCs after HNF1β interference.

To further identify the HNF1β‐regulated nuclear translocation of p65 NF‐κB, the cytoplasm and nucleus proteins were extracted from RPTC HNF1β NC and HNF1β KD cells after treatment with cisplatin. The cytosolic protein GAPDH and nucleic protein lamin B1 were used as internal control for Western blot analysis (Figure 2C). The translocation of phosphor‐P65 and total P65 from nucleus to cytoplasm was quantified using ImageJ software and summarized as Figure 2D. Cisplatin promoted the phosphor‐P65 and total P65 translocation by 2.69 and 3.39 times, respectively. The phosphor‐P65 and total P65 translocation in HNF1β KD cells was up‐regulated by 2.33 and 2.05 times, compared with HNF1β NC cells.

### 3.3 Suppression of p65 phosphorylation rescued RPTC apoptosis induced by HNF1β down‐regulation during cisplatin treatment

**FIGURE 3:**
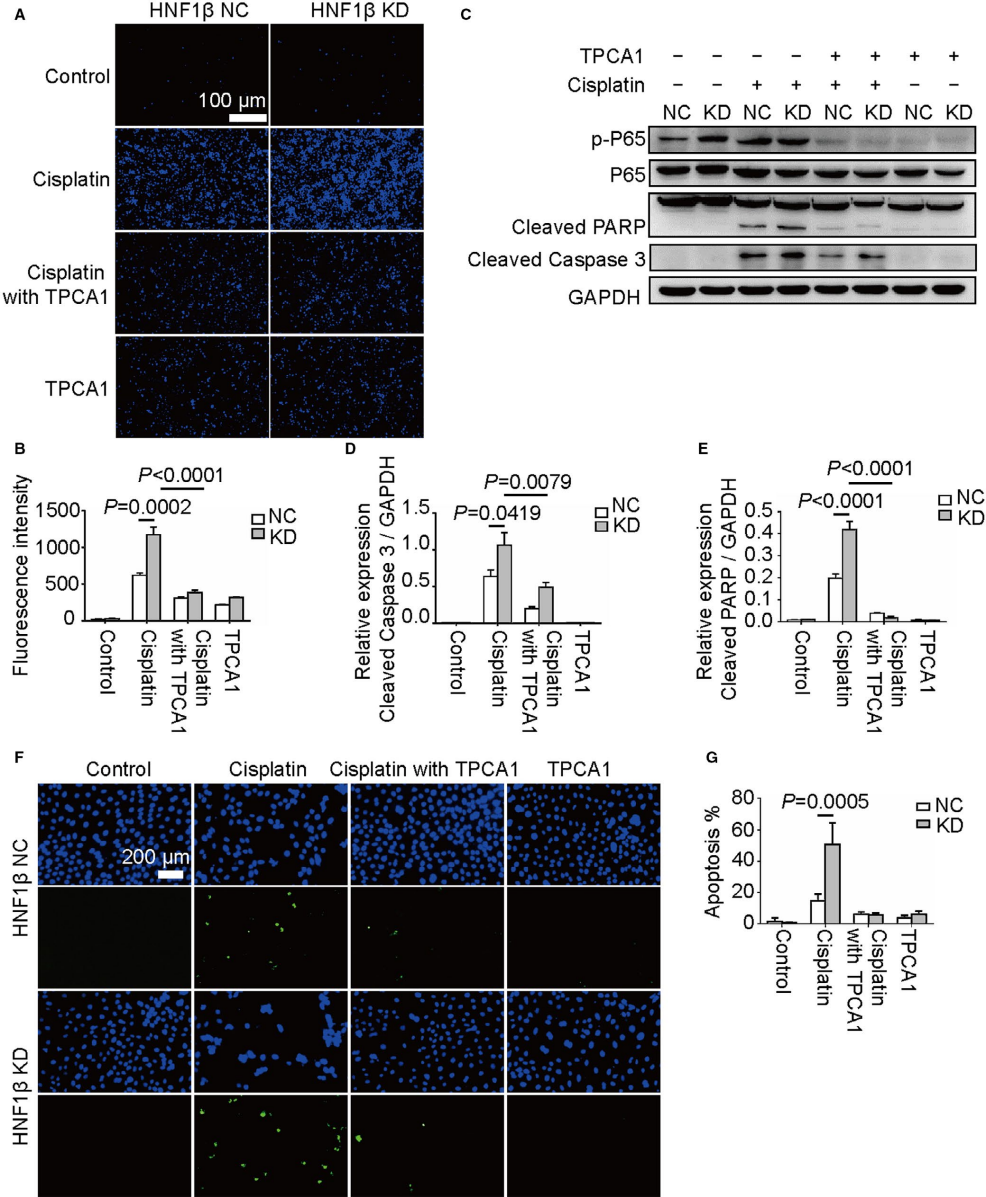
Cisplatin‐induced RPTC HNF1β NC and KD cell apoptosis affected by NF‐κB pathway inhibitor TPCA‐1. The RPTC HNF1β NC and KD cells were treated with 20 μmol/L cisplatin in combination with 50 μmol/L TPCA for 24 h. (A) Hoechst staining analysis of apoptotic cells. (B) The fluorescence intensities of blue signals in Figure 3A were quantified using ImageJ software. (C) Western blot analysis of phosphor‐P65, total P65, cleaved PARP, cleaved caspase 3 and internal control GAPDH. (D, E) Relative quantification and statistical analysis of cleaved caspase 3 and cleaved PARP using ImageJ software. (F) TUNEL staining analysis of apoptotic cells. (G) Statistical analysis of the percentage of the number of green‐positive cells in relation to the total cell number. *P* < 0.05 was considered as significant difference

To further identify the impact of NF‐κB p65 signals on cisplatin‐induced RPTC HNF1β KD cell apoptosis, the p65 nuclear translocation inhibitor TPCA‐1 was used for stimulation of RPTCs together with cisplatin. The apoptotic morphological changes in RPTCs were observed through the Hoechst staining assay (Figure 3A). HNF1β interference increased the fluorescence intensity of apoptotic cells induced by cisplatin, which were inhibited by TPCA‐1 (Figure 3B). In addition, Western blot analysis indicated that the TPCA‐1 significantly reduced the increased PARP and caspase 3 cleavages caused by cisplatin treatment and HNF1β knockdown, whereas only TPCA‐1 treatment showed no obvious influence on RPTC apoptosis (Figure 3C). After cisplatin treatment, HNF1β KD cells showed a 1.67‐fold increase in cleaved caspase 3 and 2.13‐fold increase in cleaved PARP compared with HNF1β NC cells. HNF1β KD cells showed a 69.3% reduction in cleaved caspase 3 and a 94.8% reduction in cleaved PARP after combined treatment of cisplatin and TPCA‐1, compared with individual treatment of cisplatin (Figure 3D,E). Moreover, TUNEL staining assay exhibited the enhanced apoptotic rate of RPTC HNF1β KD cells compared with HNF1β KD cells, which were all significantly reduced by TPCA‐1 (Figure 3F,G).

### 3.4 HNF1β was not colocalized with NF‐κB p65 during cisplatin‐induced RPTC apoptosis

**FIGURE 4:**
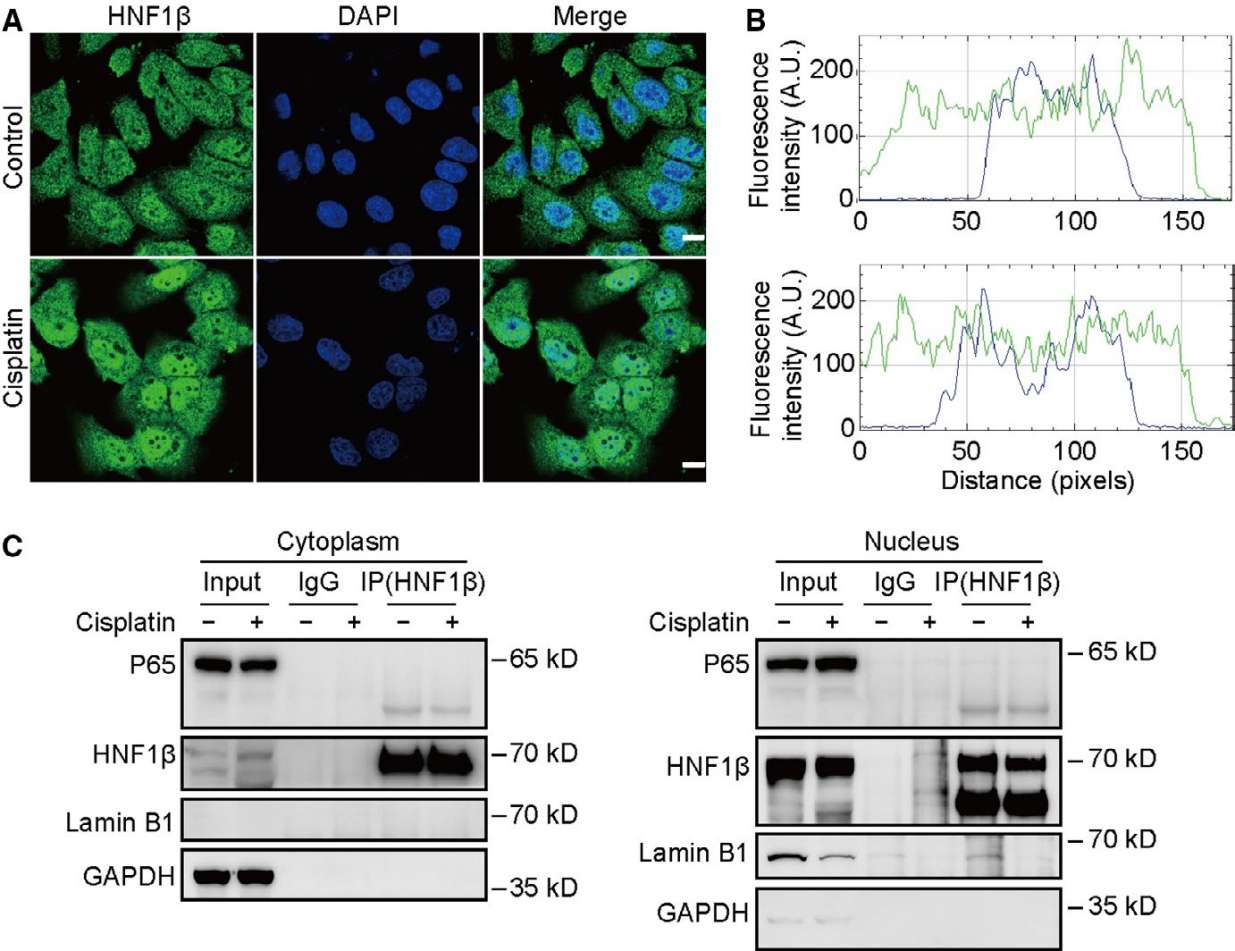
HNF1β nuclear translocation in RPTCs affected by cisplatin treatment. RPTCs were treated with or without 20 μmol/L cisplatin for 24 h and then were immunostained with anti‐HNF1β antibody and FITC‐labelled secondary antibody. The cell nucleus was stained with DAPI. (A) Fluorescence images were obtained using laser confocal microscope. Scale bars are 10 μm. (B) The fluorescence intensities of green and blue signals in Figure 4A were quantified using ImageJ software. (C) Co‐immunoprecipitation of HNF1β and P65 proteins in cytoplasm and nucleus of RPTCs treated with or without cisplatin. Lamin B1 and GAPDH were used as internal controls of nuclear and cytoplasmic proteins, respectively

As it was indicated that HNF1β was protective against cisplatin‐induced RPTC apoptosis, the nuclear translocation of HNF1β was analysed using immunofluorescent staining. As is shown in Figure 4A, cisplatin obviously promoted colocalization of HNF1β (green signals) and cell nucleus (blue signals). The quantification of fluorescence intensities of green and blue signals confirmed the results (Figure 4B).

To further analyse whether HNF1β as a nuclear transcriptional regulatory factor colocalized with P65 protein, the cytoplasm and nucleus proteins of RPTCs treated with or without cisplatin were isolated and used for co‐immunoprecipitation (IP) assay. To our surprise, whether or not treated with cisplatin in RPTCs, HNF1β did not colocalize with P65 neither in cytoplasm nor in nucleus (Figure 4C).

### 3.5 HNF1β knockdown C57BL/6 mice were successfully constructed

**FIGURE 5:**
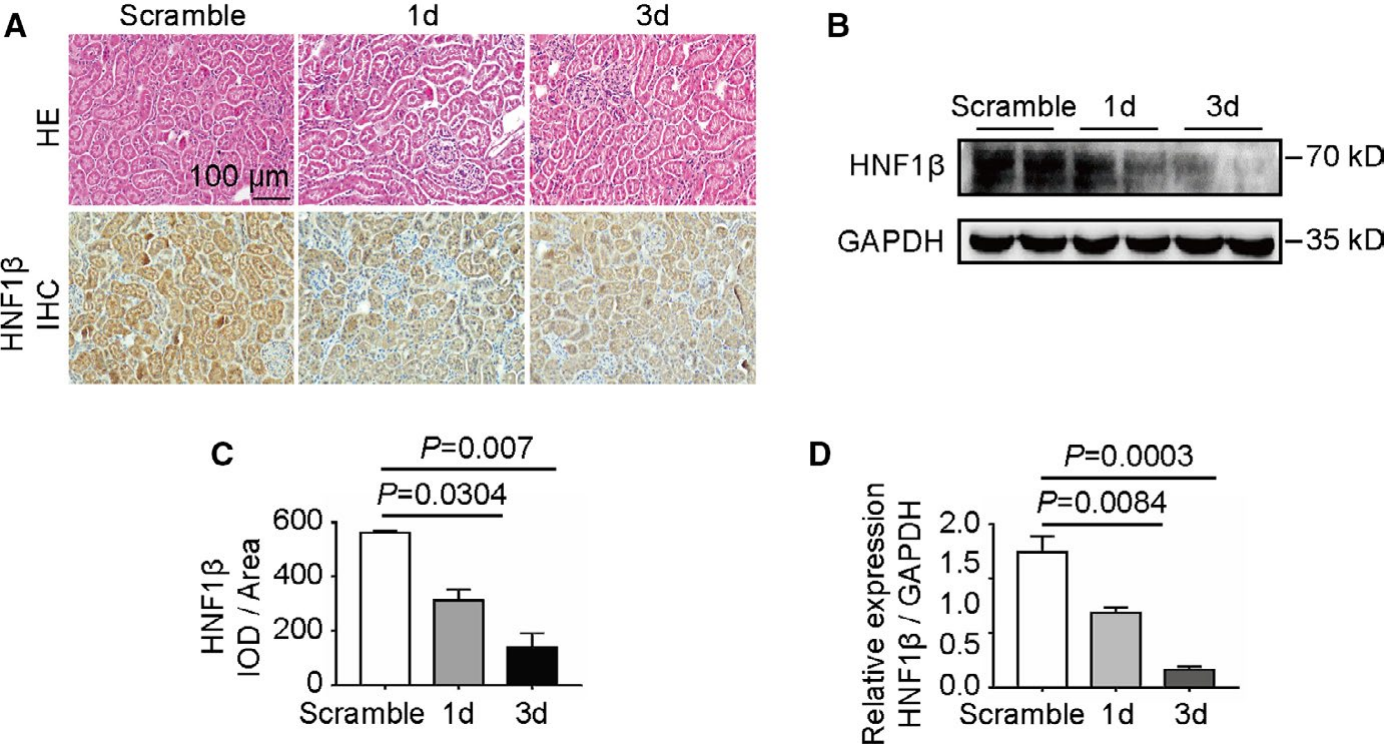
Construction of HNF1β knockdown mice. C57BL/6 mice were injected intravenously with HNF1β shRNA or scramble plasmids pre‐mixed with PEI at a nitrogen/phosphorus weight ratio (N/P ratio) of 1:2 at room temperature for 20 min. After 3 d, the renal cortex tissues were collected. (A) HE staining and IHC staining of HNF1β in renal cortex after interference with HNF1β shRNA for 1 and 3 d or scramble plasmids for 3 d. (B) Western blot analysis of HNF1β in renal cortex after interference with HNF1β shRNA for 1 and 3 d or scramble plasmids for 3 d. (C) The mean integral optical density (IOD/area) was assessed on each section from six random fields using Image‐Pro Plus 6.0 software. (D) Grey‐scale analysis of the relative expression of HNF1β protein in kidney cortex tissue in Figure 5B

To further analyse the function of HNF1β in cisplatin nephrotoxicity in vivo, HNF1β knockdown C57BL/6 mice were constructed by injecting intravenously with 40 μg HNF1β scramble shRNA and interfering shRNA. The kidney cortex after injection of scramble shRNA or interfering shRNA for 1 and 3 days was used for haematoxylin and eosin (H&E) and immunohistochemical staining (Figure 5A) and Western blot analysis (Figure 5B). As shown in Figure 5A, the tubules and glomeruli of mice were relatively intact and neatly arranged after the injection of interfering plasmid for 1 and 3 days, indicating that injection of interfering plasmid has no obvious toxic effects on renal tissue. About 44.4% and 75.1% of inhibition of HNF1β signalling were observed in renal cortex at 1 or 3 days after injection with HNF1β knockdown plasmid compared with scramble plasmid (Figure 5C). Furthermore, Western blot assay confirmed about 44.7% and 87% reduction in HNF1β expression in renal cortex at 1 or 3 days after injection with HNF1β knockdown plasmid compared with scramble plasmid (Figure 5D).

### 3.6 HNF1β ameliorated cisplatin‐induced nephrotoxicity in vivo

**FIGURE 6:**
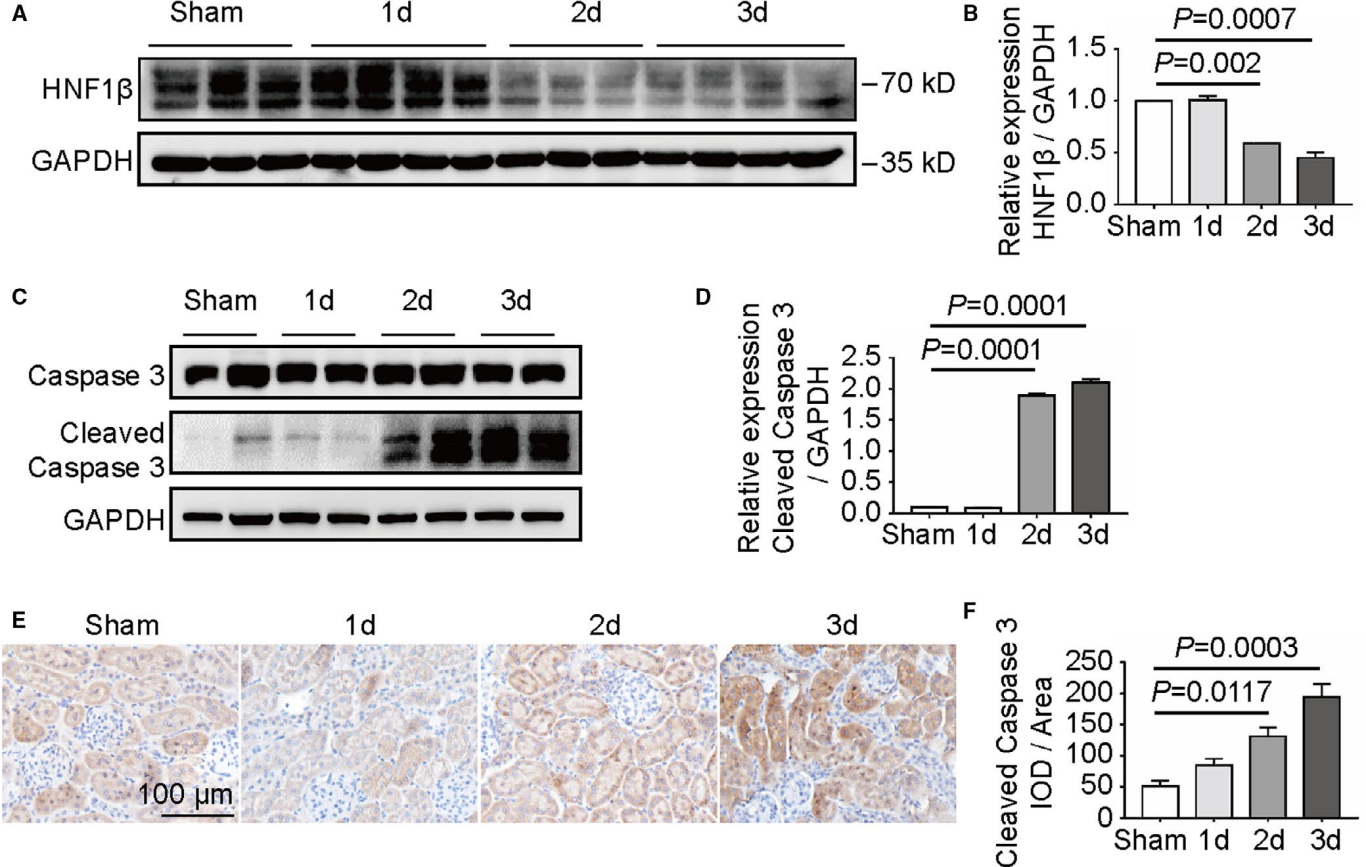
Protein expression in renal cortex tissues after treatment with cisplatin. C57BL/6 mice were injected intraperitoneally with 30 mg/kg cisplatin for 0, 1, 2 and 3 d. (A, B) Western blot analysis of HNF1β expression and caspase 3 cleavage in renal cortex. GAPDH was used as internal loading control. (C, D) The ratios of HNF1β and cleaved caspase 3 in relation to GAPDH in Figure 6A,B were quantified using ImageJ software. (E) IHC analysis of cleaved caspase 3 expression in renal cortex tissues. (F) The mean integral optical density (IOD/area) was assessed on each section from six random fields using Image‐Pro Plus 6.0 software

The expression of HNF1β was further analysed in C57BL/6 mice after cisplatin treatment for 3 days. Western blot results indicated the expression level of HNF1β was reduced after cisplatin treatment for 2 and 3 days (Figure 6A,B). The extent of caspase 3 cleavage was also enhanced after cisplatin treatment for 2 and 3 days (Figure 6C,D). The immunohistochemical staining of cleaved caspase 3 further confirmed the cisplatin‐induced nephrotoxicity in day 2 and day 3 (Figure 6E,F).

**FIGURE 7:**
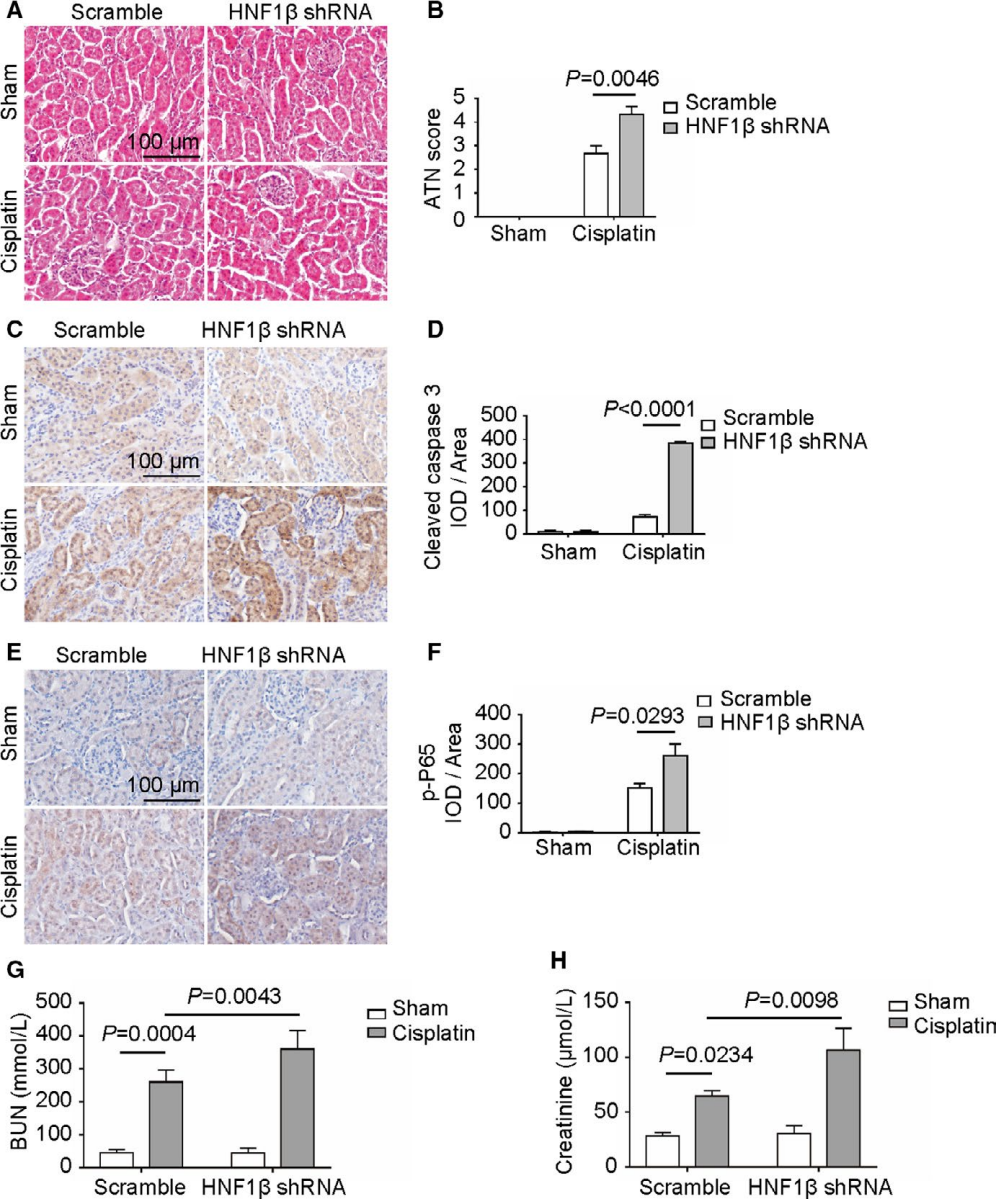
Cisplatin‐induced acute kidney injury in mice with or without interference of HNF1β. (A) HE staining of kidney cortex tissue. (B) ATN score of kidney injury shown in Figure 7A. (C) IHC staining of cleaved caspase 3 in renal cortex tissues. (D) The mean IOD/area of cleaved caspase 3 was assessed on each section from six random fields using Image‐Pro Plus 6.0 software. (E) IHC staining of phosphor‐P65 in renal cortex tissues. (F) The mean IOD/area of p‐P65 was assessed on each section from six random fields using Image‐Pro Plus 6.0 software. (G, H) The serum BUN (0580‐250; STANBIO) and creatinine (0420‐500; STANBIO) levels were quantified following Jaffe's and GLDH reactions

To further explore the functional effect of HNF1β, the HNF1β scramble and knockdown mice were treated with cisplatin for 3 days to induce acute kidney injury. HE staining indicated that the tubules in the sham groups injected with scramble or HNF1β shRNA were intact and neatly arranged. Cisplatin treatment caused obvious histological damage of kidney such as fractured tubules in control mice, which were enhanced in HNF1β knockdown mice (Figure 7A,B).

IHC staining results indicated that cleaved caspase 3 and phosphor‐p65 levels were increased in scramble mice after cisplatin treatment. Cisplatin treatment significantly increased the caspase 3 cleavage and p65 phosphorylation in HNF1β knockdown mice, compared with control mice (Figure 7C‐F). The serum urea nitrogen (BUN) and creatinine were also examined in HNF1β scramble and knockdown mice after treatment with cisplatin. Compared with control mice, cisplatin treatment significantly increased the serum levels of BUN and creatinine by 1.39 and 1.65 times, respectively (Figure 7G,H).

## 4 DISCUSSION

In this study, we found that HNF1β protects from cisplatin‐induced kidney injury both in vivo and in vitro. In addition, HNF1β may play the protective role by negatively regulate the NF‐κB signalling pathway. Cisplatin treatment promoted the nuclear translocation of HNF1β and p65 NF‐κB; however, no obvious interaction between these two proteins was observed in RPTCs either with or without cisplatin treatment.

It was surprising for us to observe the down‐regulation of HNF1β either in RPTCs or in C57BL/6 mice after the treatment of cisplatin. Then, we compared the HNF1β expression levels in different time‐points, and we found the HNF1β was induced in RPTCs by cisplatin after treatment for 6‐8 hours and reduced at 24 hours (data not shown). Moreover, the accumulation of HNF1β in nucleus after cisplatin treatment was observed, indicating that the HNF1β activation events may include nuclear translocation and regulating downstream target gene expression. HNF1β expression was reported to be induced after ischaemia/reperfusion surgery in rat for 3‐12 hours [16]. HNF1β was up‐regulated from 1 to 24 hours after hypoxia stimulation in kidney proximal tubular HK2 (human kidney 2) cells and was down‐regulated under prolonged 1% oxygen treatment [15]. The expression of HNF1β was also down‐regulated in cystic kidneys [18]. Taken together, the results demonstrated that HNF1β expression was time‐dependent and quite possible to be reduced after long‐term stimulation. In addition, the transcriptional activation of HNF1β may largely depend on the nuclear translocation.

NF‐κB activation triggers the release of cytochrome c from mitochondria, and stimulates the intrinsic apoptotic pathway, whereas P53 mainly activates extrinsic apoptotic pathway.^23^, ^24^ Upon cisplatin stimulation, HNF1β reduced the phosphorylation of p65 NF‐κB at serine‐536 but not p53, indicating that HNF1β may affect cisplatin nephrotoxicity through regulating the intrinsic apoptotic pathway.

HNF1α is the other member of HNF1 transcription factor family. HNF1α nuclear translocation was inhibited during C2‐ceramide–induced hepatocyte injury.^25^ In addition, HNF1α increased the p65 NF‐κB expression and nuclear accumulation, leading to NF‐κB signalling activation.^26^ However, in our study, HNF1β nuclear translocation was enhanced after cisplatin treatment and the NF‐κB signalling was increased after HNF1β interference, suggesting that HNF1β may regulate cell apoptosis in the opposite manner of HNF1α.

Moreover, in addition to NF‐κB nuclear translocation, the NFAT5 (nuclear factor of activated T cells‐5) was also reported to translocate from cytoplasm to nucleus during ultraviolet B irradiation–stimulating human lens epithelial cells. The interaction between NFAT5 and NF‐κB p65 subunit was also increased.^27^ Thus, NFAT5 may also be the potential downstream target of HNF1β. In this study, we did not observe the colocalization of HNF1β and NF‐κB p65 subunit neither in cytoplasm nor in nucleus, suggesting the HNF1β may indirectly modulate NF‐κB P65 signalling pathway.

In our previous study, blockade of NF‐κB decreased cisplatin‐induced microRNA‐375 expression and further increased HNF1β activity,^17^ suggesting NF‐κB can negatively regulate HNF1β activity. In this study, we confirmed that HNF1β can influence NF‐κB signalling pathway in turn, indicating that there is a mechanism of negative feedback regulation of apoptosis pathway mediated by HNF1β/NF‐κB.

In conclusion, the inhibition of HNF1β induced by cisplatin contributes to nephrotoxicity either in vitro or in vivo. HNF1β and NF‐κB signalling pathway can indirectly regulate each other and play important roles in cisplatin‐induced acute kidney injury.

## CONFLICT OF INTEREST

The authors confirm that there are no conflicts of interest.

## AUTHOR CONTRIBUTIONS

**Yan Zhang:** Formal analysis (equal); Investigation (equal); Writing‐original draft (equal). **Jielu Hao:** Formal analysis (supporting); Funding acquisition (equal); Investigation (supporting). **Zijun Du:** Investigation (equal). **Peiyao Li:** Investigation (equal). **Jinghua Hu:** Writing‐original draft (equal). **Mengna Ruan:** Writing‐original draft (equal). **Shulian Li:** Writing‐original draft (equal). **Yuanfang Ma:** Supervision (supporting). **Qiang Lou:** Conceptualization (lead); Formal analysis (equal); Funding acquisition (lead); Investigation (equal); Project administration (lead); Supervision (lead); Writing‐review & editing (lead).

## Data Availability

The data that support the findings of this study are available from the corresponding author upon reasonable request.
